# MM. Medicus and Physiologus on the Development of Intestinal Worms

**Published:** 1853-09

**Authors:** 


*MM. Medicus and Physiologus on the Development of Intestinal Worms.* The following clever and amusing summary of our present knowledge upon the development of intestinal worms, which forms a part of one of the *Colloquia de omnibus rebus*, will supply a useful wrinkle to those who have not been fortunate enough to hear or read the lectures of Professor Owen, and an agreeable reminder to those who have been so fortunate.

*Medicus.* But what do we really know of the production or reproduction of tænia?

*Physiologus.* If you had read a memoir which I have been lately reading, you would have known something about it. The author maintains that intestinal worms are neither more nor less than a more advanced stage of development of the *cysticerci* that infest the flesh of animals used as food. He has traced the tape-worm of the cat to the *cysticercus* of the mouse.

*Medicus.* But how will that account for this tape-worm forming in man, who roasts, boils, broils, or fries all his cysticerci before making a meal of them?

*Physiologus.* Nevertheless, this memoir appears to me an important addition to our knowledge of the development of intestinal worms Among the authors on this subject Professor Siebold was, I believe, the first to state from observation that the *cysticercus fascioluris* found in the liver of the mouse reaches its final state of development in the intestines of the cat, and is there transformed into the *Tænia crassicollis.* This statement was confirmed by a series of observations by Dr. Henry Nelson, who, in his thesis, presented to the University of Edinburgh, in 1850, carefully traced and figured the various stages through which the tape-worm of the cat passes. The reproductiveness of the animal exceeds even that of fishes, for each joint appears to contain 125,000 ova, which will give about twelve and a half millions for the entire worm The minute ova, thus passing off in incalculable numbers with the feces, enter the body of the mouse, mixed with its food or drink, or through the intervention of its furry coat, to which they adhere, and which it is in the habit of licking. It does not precisely appear how they pass from its alimentary canal into its liver, but most probably this is through the medium of the blood. Arrived in the liver, they are swallowed by the cat, and in its alimentary canal are converted into tape-worms—the *Tænia crassicollis.*

*Medicus.* It is easy enough to see how the mouse's cysticercus may become a feline tænia, but not conversely how the cat's tape-worm produces the cysticercus of the mouse. Do you think it in the slightest degree probable that so cleanly a creature, and so dainty a feeder, as the mouse, would eat cat's dirt, or "dip his whiskers or his tail in" such an abomination?

*Physiologus.* No; but the dirt may be converted into dust so impalpable as to escape the observation of the most fastidious of mice.

*Medicus.* A dried-up ovum is not more likely to thrive than a roasted cysticercus.

*Physiologus.* I don't admit the analogy between objects so dissimilar in size as a cysticercus half an inch long, and the ovum of a tænia 1-3000 of an inch in diameter. But, at any rate, we have now got a cat's tape-worm made out of a mouse's cysticercus. This result has been very lately extended to the tape-worms of other animals by Dr. Küchenmeister, (Prag. Vierteljahrschrift, 1852, i. 126.) He fed dogs and cats upon parts of animals which contained different kinds of cysticerci, and subsequently found tape-worms in their intestinal canal at various stages of development, according to the interval allowed to elapse after the cysticerci were devoured. Every precaution seems to have been used in these experiments, one of which may be cited. An old dog was frequently purged, for a period of six or eight weeks, with castor oil, to insure that no tape-worms should be present. He then ate food containing ten cysticerci; in seven days, ten more; and in six days more, several others, which were not counted. Nine days afterwards the dog was killed; and there were found in the intestines thirty-five tape-worms, varying from a sixth of an inch in length, and with scarcely visible joints, to well developed worms of 160 joints, and fifteen inches long. Similar results were obtained with cats. Dr. Küchenmeister, however, could not find any tape-worms in dogs fed with the liver of the mouse; but when he fed cats with the same liver, the intestines contained the *Tænia crassicollis.* It would appear, therefore, that each species of cysticercus requires a certain habitat, a peculiar animal, for its development. It has been rendered highly probable that the *Cysticercus fasciolaris* of the mouse is transformed into the *Tænia crassicollis* of the cat, the *C. tenuicollis* of the squirrel and ruminant animals into the *T. serrata* so common in the dog, and the *C. pisiformis* of the hare and rabbit into the *T. crassiceps* of the fox.

As for the human tape-worms, Eschricht has rendered it highly probable that the *Bothriocephalus latus*, which occurs in man in Russia and some other countries, is the farther development of a species of *Ligula*, which infests the flesh of the dorse and other fishes of the Northern seas. The origin of the *Tænia solium*, the only tape-worm met with in this country, is most probably the *Cyticercus cellulosæ* which inhabits the flesh of various animals, and especially the pig and sheep. Dr. Nelson, at least, points out the following reason for this opinion. In both, the neck is attenuated. In both, the body gradually increases in size downwards. In both, the head is similar, the figures given of them by Bremser being identical; and, in particular, both heads have a double circle of hooks; and although the *Tænia solium* is sometimes seen without hooks, Bremser has proved this to be the result of age, one row dropping off first, and then also the other.

It is not so difficult as you [to Medicus] suppose to account for the admission of living cysticerci into the human intestines. Passing by the remarkable fact that tape-worm is nowhere so common in the human subject as in Abyssinia, where meat is habitually eaten in the raw state—and also setting aside as doubtful the alleged power of the cysticerci to resist an elevated temperature, because in that case tape-worm should be as common in Britain as Abyssinia—we know that there are many people who prefer their meat in a very underdone state, and few are on their guard against a partial error of the cook in this respect.

In regard to the importance of obtaining the tape-worm's head, when the worm is expelled by remedies, it must of course be allowed to be extremely difficult to discover in the feces so minute an object as the head of the *Tænia solium*. But this does not affect the importance of its expulsion as a guarantee of a radical cure.

Late anatomical researches rather confirm the prepossessions of physicians in favor of getting a sight of the head, if possible. Thus, Van Benedin has shown that the head is the part from which all the joints are thrown off by gemmiferous reproduction; those formed first being pushed downwards by the formation of others, and being farther developed as they descend. He considers the tape-worm as a compound fluke-worm, the whole consisting of three stages or states of growth: 1st, the cystic head, (scolex;) 2d, the compound tape-worm, (strobila;) 3d, the separated joints, (proglottis.) And Dr. Nelson has ascertained that the four suckers of the head of the worm contain each a canal, which afterwards unite to form the two lateral canals of its body; and he describes the manner of feeding and propulsion of the contents of these canals from the head to the tail. The head is therefore important on two accounts: 1st, as the means by which the animal is nourished; and 2ndly, as the basis on which the joints are produced. Van Benedin, indeed, supposes that when the tail-joints separate, as they often do, they may themselves go on developing, and become a species of fluke or *Distoma.* But this view is far from being established.

*Editor.* It is to be hoped that these results will stand the test of time. It is gratifying to see the dry anatomical researches of Rudolphi, Bremser, and others, giving forth useful results at last.

*Physiologus.* Those foolish people in our profession who so eagerly search only for what they call practical information, may rest assured that, in the long run, the cultivation of pure science is the surest and best way of arriving at it. No one could have expected, when Galvani made the limbs of a decapitated frog caper, and when practical men got no answer to their question, *cui bono*—that England and France would one day be united by a galvanic telegraph. Nor could it have been imagined that the apparently barren labors of Goetz, Rudolphi, and others, would lead to the important practical fact that tape-worm is caused by feeding on measly meat and fish out of season, or by indulging in the heathenish custom of devouring half-raw flesh.—*Edinburgh Monthly Journal.*

